# Modelling Sorption Thermodynamics and Mass Transport of n-Hexane in a Propylene-Ethylene Elastomer

**DOI:** 10.3390/polym13071157

**Published:** 2021-04-04

**Authors:** Daniele Tammaro, Lorenzo Lombardi, Giuseppe Scherillo, Ernesto Di Maio, Navanshu Ahuja, Giuseppe Mensitieri

**Affiliations:** 1 Department of Chemical, Materials and Production Engineering, University of Naples Federico II, Piazzale Tecchio 80, 80125 Naples, Italy; daniele.tammaro@unina.it (D.T.); lorenzo.lombardi@unina.it (L.L.); gscheril@unina.it (G.S.); edimaio@unina.it (E.D.M.); 2 UdR of Naples, INSTM (National Interuniversity Consortium of Materials Science and Technology), Piazzale Tecchio 80, 80125 Naples, Italy; 3 Sulzer Chemtech Ltd., Else-Züblinstr. 11, 8404 Winterthur, Switzerland; navanshu.ahuja@sulzer.com

**Keywords:** polyolefin elastomer, n-hexane, sorption thermodynamics, lattice fluid theory, diffusivity

## Abstract

Optimization of post polymerization processes of polyolefin elastomers (POE) involving solvents is of considerable industrial interest. To this aim, experimental determination and theoretical interpretation of the thermodynamics and mass transport properties of POE-solvent mixtures is relevant. Sorption behavior of n-hexane vapor in a commercial propylene-ethylene elastomer (V8880 Vistamaxx^TM^ from ExxonMobil, Machelen, Belgium) is addressed here, determining experimentally the sorption isotherms at temperatures ranging from 115 to 140 °C and pressure values of n-hexane vapor up to 1 atm. Sorption isotherms have been interpreted using a Non Random Lattice Fluid (NRLF) Equation of State model retrieving, from data fitting, the value of the binary interaction parameter for the n-hexane/V8880 system. Both the cases of temperature-independent and of temperature-dependent binary interaction parameter have been considered. Sorption kinetics was also investigated at different pressures and has been interpreted using a Fick’s model determining values of the mutual diffusivity as a function of temperature and of n-hexane/V8880 mixture composition. From these values, n-hexane intra-diffusion coefficient has been calculated interpreting its dependence on mixture concentration and temperature by a semi-empiric model based on free volume arguments.

## 1. Introduction

The advancements of metallocene catalyst technology have accelerated the growth and development of polyolefin elastomers (POE) with a finely controlled structure-property characteristics [1]. These elastomers, belonging to the broader class of thermoplastic elastomers (TPE), have been increasingly in use due to their superior mechanical and thermal properties, versatility of use and recyclability [2]. Solution polymerization is one of the key processes to produce polypropylene (PP) based elastomers. The production of propylene-based polyolefins with a variety of properties has continuously grown, since the discovery of Ziegler–Natta catalysts, boosted by a rapid development of catalyst technology combined with polymerization process innovation. Among them, a relevant class is that of propylene-ethylene copolymers, that are semi-crystalline performance polymers with tunable amorphous content, mechanical and optical properties. They are economical, recyclable and can be designed for a specific application (e.g., adhesives, packaging, etc.).

Thermodynamics of mixtures of POE and alkane/alkenes hydrocarbons system are of huge interest for many industrial applications, like designing polymerization processes, polymer purification technology, and optimizing batch or continuous polymer foaming processes. For instance, the role of solvent concentration in the mentioned processes is fundamental for the following operations:

- Polymerization. The monomer concentration, which can be dissolved in the polymer, determines the concentration at the active site, affecting polymerization rate and molecular weight distribution [3].
- Polymer foaming. The blowing agent concentration, which can be solubilized in the polymer, affects the final expansion ratio, the cellular morphology, and the final foamed shape [4].
- Separation technology: the maximum degree of separation, i.e., solvent concentration at equilibrium that may be obtained for a given system, is the most important parameter of interest to optimize the equipment design, while controlling the strict levels of volatile organic compounds (VOCs) in the final product [5].

From a practical viewpoint, the accurate knowledge of the thermodynamics of such polymer–solvent mixtures can be extremely beneficial to optimally model and design polymer plants, control stream compositions going into upstream units and importantly, abide the strict measures on final product quality, thereby creating a significant industrial impact. Furthermore, such analysis can be extended to systems with similar characteristics, providing support to the ongoing research and contribute to the innovation in the field of polymer processing.

Understanding thermodynamics of mixtures of long-chain molecules with short, non-polar hydrocarbon molecules, requires sophisticated experiments to provide high quality data and reliable thermodynamic models to explain their complex non-ideal behavior. Sorption thermodynamics has been the center of research for many decades, aimed at describing phase equilibria of polyolefins with several small molecules, involved in different polymer processing applications.

Even though several sorption studies of multiple gases in polymers are mentioned in the literature (e.g., butane in PET, xylene/nonane in Polystyrene, N_2_/CO_2_ in PP, non-polar organic solvents with Poly-isobutylene, PLA, etc.) [6], in the case of POE thermodynamics studies are rare. In effect, most of the experimental results and theoretical approaches reported in the literature for POE are restricted to the study of the solubility and transport of gases [7] or to very low polymer content [5] or at temperatures below the polymer melting temperature [4], while investigations focused on sorption of vapors, particularly vapors of alkanes, are scarce [8]. The lack of reliable thermodynamics data of POE/alkane system at high polymer content and temperature makes the optimization of the aforementioned industrial processes and the validation of models difficult.

Elaboration of models for the thermodynamics of polymer containing mixtures, adequate for system description over a wide range of pressure and temperature conditions, is still an active and fascinating research area. To this end, a large number of thermodynamic approaches based on statistical thermodynamics have been built rooted on the lattice models developed by Guggenheim [9] and Flory [10] for complex fluids, including polymers. A relevant example is the lattice fluid (LF) theory proposed by Sanchez and Lacombe (SL) [11,12,13], which was successfully adopted to deal with polymer solutions starting from 1976. Since then, other models, still based on a lattice fluid framework, were introduced with improved performances in terms of explicit account of the non-random distribution of molecular species and free volume, as well as of presence of strong specific interactions possibly established between neighboring molecules, such as hydrogen bonding [14,15,16,17,18]. SL and the following improved LF approaches provide both an expression for the Equation of State (EoS) of pure fluids and mixtures as well as for the chemical potentials of the components of a mixture and can be applied to fluids over an extended range of external conditions, encompassing liquids, vapors, gases, supercritical fluids, amorphous and glassy polymers, homogeneous as well as inhomogeneous systems, complex aqueous systems, associated polymer mixtures, rubbers, and gels. A relatively recent development of this kind is the Non-Random-Hydrogen-Bonding model (NRHB) proposed by Panayiotou et al. [17,18], a compressible lattice fluid EoS theory that, besides the mean-field interactions, accounts also for the presence of specific interactions (e.g., hydrogen bonding, Lewis acid/Lewis base interactions) and for non-random distribution of components and of free volume in pure compounds [17] as well as in their mixtures [18].

A lot of research efforts have been devoted in making EoS models able to accurately describe the polymer solvent phase equilibrium. An EoS can describe the pure component, as well as the mixture properties. Solubility of multiple gases like N_2_, H_2_, He, CO_2_, in polyolefins have been modelled with different EoSs. However, models to accurately predict equilibrium sorption thermodynamics of small chain hydrocarbon in POEs have not been thoroughly tested, especially in the concentrated polymer regime [6]. In addition, the model parameters for POE are not readily available.

In the present contribution we address the case of n-hexane sorption in a commercial POE made of isotactic propylene repeating units with random ethylene distribution, produced by ExxonMobil under the trade name Vistamaxx 8880 (V8880). The analysis of this system is of interest for the correct identification of optimal conditions in the processing of this polymer. Sorption isotherms of n-hexane vapor at several temperatures have been determined by gravimetry and data were interpreted using a Non Random Lattice Fluid (NRLF) model that is essentially analogous to the NRHB model but without the terms accounting for specific interactions, which have not to be considered here in view of the chemical structure of the polymer (polyolefin) and of the penetrant (n-hexane). The analysis was performed at low relative pressures of n-hexane vapor since the main interest was for tailoring processing conditions at low n-hexane concentration conditions. Parameters of the NRLF model for the pure components were determined by fitting with the model respectively the experimental PVT behavior of the polymer and the density and vapor pressure data at vapor-liquid equilibrium available in the literature for n-hexane. Experimental sorption isotherms were then fitted through the NRLF model to retrieve the value of the binary interaction parameter for the V8880-n-hexane mixture. Mass transport properties were also investigated determining experimentally the mutual diffusivity of the V8880/n-hexane system at several temperatures and concentrations. From these values, applying a model based on free-volume arguments, was estimated the n-hexane intra-diffusion coefficient in V8880, whose dependence on temperature and mixture composition was interpreted using a semi-empirical model.

## 2. Theoretical Background

### 2.1. Modeling Sorption Thermodynamics by NRLF Approach

An evolution of classical compressible LF theories used to describe the thermodynamics of amorphous rubbery polymer–penetrant mixtures, consisted in introducing modifications to the SL theory by accounting for the for non-random distribution of components and of free volume. The NRLF approach adopted here belongs to this class of models and is derived from the NRHB model [17,18,19,20,21] by dropping out the terms related to specific interactions. We address here only the specific case of a binary system made of a polymer and a low molecular weight penetrant.

Analogously to the NRHB model, the thermodynamic behavior of a pure component is described using only three characteristic scaling parameters, i.e., $v_{i,sp,0}^{*}$, $\varepsilon_{i,h}^{*}$ and $\varepsilon_{i,s}^{*}$. The first one, $v_{i,sp,0}^{*}$ (cm^3^ × g^−1^), appears in the following expression [20,22] adopted to calculate the closed packed specific volume of component *i*, $v_{i,sp}^{*}$ (cm^3^ × g^−1^):

(1) $$v_{i,sp}^{*}=v_{i,sp,0}^{*}+\left(T-298.15\right)v_{sp,1}^{*}$$

where $v_{sp,1}^{*}$ (cm^3^ × g^−1^ × K^−1^) is a constant for a given homologous series of compounds [23,24,25,26,27,28,29] and it is set equal to −0.412·10^−3^ cm^3^ × g^−1^ × K^−1^ for non-aromatic hydrocarbons, −0.310·10^−3^ cm^3^ × g^−1^ × K^−1^ for alcohols, −0.240·10^−3^ cm^3^ × g^−1^ × K^−1^ for acetates, −0.300·10^−3^ cm^3^ × g^−1^ × K^−1^ for water, and 0.150·10^−3^ cm^3^ × g^−1^ × K^−1^ for all the other fluids [28,29]. Finally, *T* represents the temperature (expressed in Kelvin). 

In the case of a polymeric compound, Equation (1) is modified so that it takes also into account for the possible pressure dependence, becoming:

(2) $$v_{i,sp}^{*}=v_{i,sp,0}^{*}+\left(T-298.15\right)v_{sp,1}^{*}-0.135\cdot 10^{-3}P$$

where *P* represents the pressure (expressed in this equation in MPa).

The other two energy parameters, $\varepsilon_{i,h}^{*}$ (J × mol^−1^) and $\varepsilon_{i,s}^{*}$ (J × mol^−1^ × K^−1^), which represent, respectively, the enthalpic and entropic terms, are needed to calculate the average mean field interaction energy per molecule of component *i*, $\varepsilon_{i}^{*}$, that is expressed as [15,17]:

(3) $$\varepsilon_{i}^{*}=\varepsilon_{i,h}^{*}+\left(T-298.15\right)\varepsilon_{i,s}^{*}$$

Notably, the volume occupied by a cell of a molecule of species *i*, $v_{i\;}^{*}$, is assumed to take the universal value of 9.75/*N_AV_* cm^3^/molecule, with *N_AV_* representing the Avogadro number. Consistently, in the case of a mixture, the molar volume of lattice cells, indicated as v*, takes the same value, independently of concentration. This is a relevant point since this assumption of a constant universal value for $v_{i\;}^{*}$ and v* guarantees the thermodynamic consistency of the thermodynamic model [22], which is, instead, not granted in other lattice fluid theories (see for example the case of the SL model, as discussed in [22]). From this set of parameters, the number of lattice cells occupied by one molecule of species *i*, *r_i_*, can be calculated [18,22] using the following expression:

(4) $$r_{i}=\frac{M_{w,i}v_{i,sp}^{*}}{v_{i}^{*}}$$

where $M_{w,i}$ is the molecular weight of component *i*.

The values of the three model parameters for the pure component *i*, i.e., $v_{i,sp,0}^{*}$, $\varepsilon_{i,h}^{*}$ and $\varepsilon_{i,s}^{*}$, are generally retrieved from vapor pressure and/or volumetric properties for the case of a pure component with a low molecular weight while, in the case of a polymer, are retrieved from PVT data in the melt state.

An additive parameter is the so-called molecular shape factor, *s_i_*, defined as the ratio between the number of lattice external contacts per molecule of component *i*, *q_i_*, and *r_i_*. This parameter can be either evaluated using the UNIFAC group contribution model [30] or can be retrieved from fitting procedures of experimental data, along with the three characteristics scaling parameters. In Section 2 of the Supplementary Materials document the procedure to estimate these four parameters for the case of n-hexane and of the V8880 polymer is reported.

The relevant model equations (i.e., the expression of Gibbs energy and volumetric EoS) for pure components are expressed in terms of dimensionless reduced variables, i.e., reduced temperature, ${\tilde{T}}_{i}$ reduced pressure ${\tilde{P}}_{i}$ and reduced density ${\tilde{\rho }}_{i}$:

(5) $${\tilde{T}}_{i}=\frac{T}{T_{i}^{*}};\;\;\;{\tilde{P}}_{i}=\frac{P}{P_{i}^{*}};\;\;\;{\tilde{\rho }}_{i}=\frac{\rho }{\rho_{i}^{*}}=\frac{1}{{\tilde{v}}_{i}}$$

The corresponding normalizing factors for the temperature *T*, pressure *P* and density *ρ*, i.e., T*, P*, and ρ*, are interrelated via the following expressions [17]:

(6) $$\rho_{i}^{*}=\frac{M_{w,i}}{r_{i}v^{*}}$$

(7) $$\varepsilon_{i}^{*}=kT_{i}^{*}=P_{i}^{*}v^{*}$$

where *k* is the Boltzmann constant. 

In the following, we will indicate with subscript “1” the quantities referred to the low molecular weight penetrant and with subscript “2” those related to the polymer. For the case of a binary mixture of components “1” and “2”, the average mean field interaction energy per molecule, ε*, is obtained through the following mixing rule:

(8) $$\varepsilon^{*}=\theta_{1}^{2}\varepsilon_{1}^{*}+2\theta_{1}\theta_{2}\varepsilon_{12}^{*}+\theta_{2}^{2}\varepsilon_{2}^{*}$$

where *θ_1_* and *θ_2_* are the so-called surface contact fractions [18] which depend on concentration and

(9) $$\varepsilon_{12}^{*}=\;\left(1-k_{12}\right)\sqrt{\varepsilon_{1}^{*}\varepsilon_{1}^{*}}\;$$

where the binary interaction parameter, *k*_12_, measures the departure of mean field interaction energy from the value provided by the geometric mixing rule. Analogously, in a binary mixture, parameters *r* and *q* are calculated using the following simple mixing rules:

(10) $$r=r_{1}x_{1}+r_{2}x_{2}$$

(11) $$q=q_{1}x_{1}+q_{2}x_{2}$$

and so

(12) s=qr

where *x_i_* is the molar fraction of component *i*.

As for pure components, also for a binary mixture, dimensionless reduced variables, i.e., reduced temperature $\tilde{T}$, reduced pressure $\tilde{P}$ and reduced density $\tilde{\rho }$ [18], can be defined as follows:

(13) $$\tilde{T}=\frac{T}{T^{*}}=\frac{kT}{\varepsilon^{*}}$$

(14) $$\tilde{P}=\frac{P}{P^{*}}=\frac{Pv^{*}}{kT^{*}}$$

(15) $$\tilde{\rho }=\frac{1}{\tilde{v}}=\frac{Nrv^{*}}{V}$$

where *V* is the volume of the mixture and *N* is the total number of molecules in the mixture. It is worth to recall that, also in the case of a mixture, $v^{*}$ is assumed to take the universal value of 9.75/*N_AV_* cm^3^/molecule.

The NRLF model provides the dimensionless expressions for the EoS of both the pure components and their mixtures, that take the same form in terms of reduced variables [17,18]:

(16) $$\tilde{P}+\tilde{T}\left[\ln\left(1-\tilde{\rho }\right)-\tilde{\rho }\left(\sum_{i}\varphi_{i}\frac{l_{i}}{r_{i}}\right)-\frac{z}{2}\ln\left(1-\tilde{\rho }+\frac{q}{r}\tilde{\rho }\right)+\frac{z}{2}\ln\Gamma_{00}\right]=0$$

where $l_{i}=\left(z/2\right)\left(r_{i}-q_{i}\right)-\left(r_{i}-1\right)$ and *z* is the coordination number of the lattice in which the molecules are assumed to be arranged, $\varphi_{i}$ represents the “close packed” volumetric fraction of species *i*, and *q*, defined by Equation (11), represents the average number of lattice contacts per molecule in the mixture. In the NRLF model, the state variables $\Gamma_{ij}$ represent the multiplicative corrective factors accounting for the non-randomness of contacts among molecular sites of species *j* and molecular sites of species *i* within the lattice (*i*,*j* = 0,1,2; in particular, index equal to 0 stands for the empty cells of lattice). Their values can be obtained by solving a set of equations, obtained by minimizing Gibbs free energy as a function of number of different kinds of lattice fluid contacts and by imposing material balance expressions for the lattice fluid contacts [17,18]. In particular, $\Gamma_{00}$ accounts for non-random distribution of free volume.

Occurrence of phase equilibrium between a binary polymer–penetrant mixture and the pure penetrant in a vapor or liquid state implies the equality of the chemical potentials of the penetrant in the two coexisting phases:

(17) $$\mu_{1}^{V}=\mu_{1}^{P}$$

where $\mu_{1}^{V}$ represents the molar chemical potential of penetrant in the pure vapor/liquid phase while $\mu_{1}^{P}$ represents that in the polymer–penetrant mixture. In the case of high molecular weight polymers, as is the case at hand, it is assumed that macromolecules are insoluble in the pure penetrant vapor phase in contact with it. As a consequence, no expression equating the chemical potentials of the polymer in the two phases at equilibrium is imposed and only Equation (17) rules the phase equilibrium. The expression of the chemical potential of the penetrant within the polymer–penetrant phase takes the following dimensionless form [18]:

(18) $$\begin{array}{l} \frac{\mu_{1}^{P}}{RT}=\ln\frac{\varphi_{1}}{\omega_{1}r_{1}}-r_{1}\sum_{j=1}^{2}\frac{\varphi_{j}l_{j}}{r_{j}}+\ln\tilde{\rho }+r_{1}\left(\tilde{v}-1\right)\ln\left(1-\tilde{\rho }\right)-\frac{z}{2}r_{1}\left(\tilde{v}-1+\frac{q_{1}}{r_{1}}\right)\ln\left(1-\tilde{\rho }+\frac{q}{r}\tilde{\rho }\right)+\\ \frac{zq_{1}}{2}\left[\ln\Gamma_{11}+\frac{r_{1}}{q_{1}}\left(\tilde{v}-1\right)\ln\Gamma_{00}\right]+r_{1}\frac{\tilde{P}\tilde{v}}{\tilde{T}}-\frac{q_{1}}{\tilde{T}} \end{array}$$

where *R* represents the universal constant of gases and, $\omega_{i}$ represents a characteristic quantity that accounts for the flexibility and symmetry of molecule of kind *i*, and it is defined in refs. [16,17]. This expression must be coupled with the EoS reported before (Equation (16)).

The expressions of the EoS and of the chemical potential for pure penetrant in the vapor or liquid state can be obtained respectively from Equations (16) and (18) by setting $\varphi_{1}=1$ and the number of components in the summation equal to 1.

The NRLF model described above is suitable to deal with the sorption thermodynamics of low molecular weight penetrants in amorphous rubbery polymers. This theoretical approach has been considered appropriate for the interpretation of sorption isotherms of n-hexane in V8880 since tests have been performed at a temperature of 115 °C and higher, at which the polymer can be safely assumed to be amorphous in view of the value of the melting temperature of the neat polymer, 97 °C (see Table S1 in the Supplementary Materials document), and of the fact that absorbed n-hexane is expected to promote a decrease of the melting temperature below this value [10].

### 2.2. Modeling Diffusive Mass Transport of n-Hexane

Mass transport of low molecular weight compounds in rubbery polymers is generally ruled by the so-called Fickian constitutive law for diffusion (see the classical reference [31]). In fact, diffusion of gases in rubbery polymers (e.g., oxygen in polyolefins) can be described by a mass balance where the mass diffusive flux is expressed by the Fick’s law with a binary (mutual) diffusivity independent of concentration of penetrant. This is known as “Ideal Fickian” behavior. In the cases of diffusion of vapors in rubbery polymers, still a Fickian constitutive expression could be used to express the mass flux, but, in general, a concentration dependent binary (mutual) diffusion coefficient is needed. This is known as “non-Ideal Fickian” behavior. In this latter case the dependence of diffusivity on concentration should be known to interpret sorption kinetics. A way to circumvent this difficulty is to consider sorption step experiments in which a relatively small increment of pressure of vapor is imposed at each step, so that a relatively small change in concentration occurs inside the polymer sample during the sorption experiment. In such a case, the diffusivity can be assumed to take a roughly constant value (i.e., an average value in the range of concentration established within the sample during the sorption step). Additionally, it is to be considered that, if the concentration of penetrant is rather high (in general above 10%), beside the mutual diffusion contribution to the mass flux, one should also consider a mass convective contribution related to the average bulk movement of the polymer–penetrant mixture as a result of interpenetration of the components.

In the case at hand, we have interpreted sorption kinetics data assuming a non-Ideal Fickian behavior. Since each sorption step was accompanied by a relatively small pressure (and, consequently, small concentration) increase, each sorption step was interpreted using the classical solution of the differential mass balance provided by a Ideal Fickian constitutive expression for mass flux [32]. Obviously, since diffusivity is expected to have some degree of dependence on concentration, one also expects to determine different values of diffusivity at each step. The determined value of binary (mutual) diffusivity can be assumed to be associated to the average value of concentration of n-hexane present within the sample (i.e., the average between the initial and final values of concentration of the step considered). In addition, in view of the relatively small values of n-hexane concentration (mass fraction values <0.1, i.e., percentage <10%), no mass convection (bulk flow) contribution has been considered in the expression for n-hexane flux. 

In the case of an Ideal Fickian behavior, experimental sorption kinetics at each pressure step can be expressed, in the case of diffusion of a penetrant in a plane sheet at a uniform initial internal concentration of penetrant and uniform and constant concentrations of the penetrant at the sample surface, by [32]:

(19) $$\frac{M_{t}}{M_{\infty }}=1-\sum_{n=0}^{\infty }\;\;\frac{8}{{\left(2n+1\right)}^{2}\pi^{2}}\exp\left[-\frac{D{\left(2n+1\right)}^{2}\pi^{2}t}{4l^{2}}\right]$$

where $M_{t}$ denotes the total amount of diffusing substance which has entered the sheet at time t, $M_{\infty }$ is the corresponding quantity after infinite time (i.e., at equilibrium) and 2*l* is the sheet thickness. It is worth noting that the mutual binary diffusion coefficient D, that is the fitting parameter, is characteristic of the polymer–penetrant couple considered. In view of the sample geometry adopted in this investigation (the sample is placed at molten state in a cylindrical pan), we can consider that we are testing a plane sheet with only one surface exposed to the penetrant vapor, the other being adherent to the surface of the pan. As a consequence, in using Equation (19), the adopted value of 2*l* is actually twice the real sheet thickness.

To gather information on the true intrinsic mobility of each component, one should consider the “intra-diffusion” coefficient of each of them. In fact, the intrinsic mobility of n-hexane in the polymer-penetrant mixture is represented by the n-hexane intra-diffusion coefficient (indicated as *D*_1_ in the present context), that reflects the mobility of n-hexane molecules in the absence of any driving force, in particular that expressed by the gradient of chemical potential. Sometimes this coefficient is also reported as self-diffusion coefficient [33] although this term is more appropriate with reference to the case of a pure component (i.e., self-diffusivity of pure n-hexane or of pure polymer, representing the intrinsic mobility of the molecules of a component in a pure state). The intra-diffusion coefficient of a penetrant tends to the value of the penetrant self-diffusion coefficient as its mass fraction in the mixture tends to 1. The same is true for the polymer, i.e., the *intra*-diffusion coefficient of polymer (indicated as *D*_2_ in the present context) tends to the value of the polymer *self*-diffusion coefficient as the mass fraction of polymer in the mixture tends to one. While the mutual diffusivity is simple to measure, the *intra*-diffusion (or *self*-diffusion) coefficient is more complex to evaluate experimentally. There are theories, however (as, for example the free volume theory of Vrentas and Duda [34,35]), providing theoretical or semi-empirical expressions for the intra-diffusion coefficients. Then, if some simplifying assumption is taken as appropriate, (i.e., the penetrant–polymer molecular friction coefficient is the geometric average of the penetrant–penetrant and polymer–polymer molecular friction coefficients [34,35]), one can express the mutual diffusivity in terms of the *intra*-diffusion coefficients of the components of the mixture. In the case of low concentration of the penetrant, a further simplification can be adopted, and *D* can be expressed only in terms of *D*_1_ [34,35] (see the following Equation (20)).

Based on these premises, we can relate the n-hexane-V8880 mutual diffusion coefficient, *D*, to the n-hexane intra-diffusion coefficient, *D*_1_, according to the following “free volume theory” expression [34,35]:

(20) $$D=\frac{D_{1}\cdot \rho_{2}\cdot {\hat{V}}_{2}\cdot \rho_{1}}{RT}{\left(\frac{\partial \mu_{1}}{\partial \rho_{1}}\right)}_{T,P}$$

where ${\hat{V}}_{2}$ represents the partial specific volume of the polymer, $\rho_{1}$ and $\rho_{2}$ represent, respectively, the density of n-hexane and of polymer (expressed in grams of n-hexane or polymer per unit volume of polymer–penetrant mixture) and $\mu_{1}$ represents the equilibrium molar chemical potential of n-hexane within the mixture at the given conditions. The values of ${\hat{V}}_{2}$ and of ${\left(\partial \mu_{1}/\partial \rho_{1}\right)}_{T,P}$ can be evaluated numerically using the NRLF model for mixtures. 

## 3. Materials and Methods

### 3.1. Materials

Details on the materials (polymer [36] and n-hexane) properties are reported in the Supplementary Materials document.

### 3.2. Charaterzation of PVT Behavior

Specific volume of the V8880 elastomer, at equilibrium conditions, has been measured as a function of temperature and pressure in the ranges 25–200 °C and 10–200 MPa, to be used for the determination of NRLF model parameters for the polymer. Description of the adopted apparatus and results of data fitting with NRLF model are reported in the SI document. 

### 3.3. Equilibrium Data for n-Hexane

Vapor pressure and equilibrium density data at liquid–vapor equilibrium for n-hexane, to be used for the determination of NRLF model parameters for n-hexane, were retrieved from thermodynamics databases. Details on the source of these data and the results of data fitting with NRLF model are reported in the SI document.

### 3.4. Gravimetric Sorption Tests

Vapor sorption experiments of n-hexane in V8880 were performed using a controlled atmosphere McBain micro-balance to determine n-hexane sorption kinetics in V8880 as well as the amount of n-hexane absorbed at equilibrium, at different values of temperature and of pressure of n-hexane vapor. The tests have been performed according to a standard procedure described in full details in [37]. Full details on the apparatus and on the experimental procedure are reported in the SI document. 

## 4. Results

Experimental sorption tests of n-hexane vapor in V8880 have been performed at 115, 122, 130, and 140 °C. At each temperature, vapor pressure of n-hexane has been increased stepwise collecting at each step sorption kinetics and the equilibrium sorption value. The range of pressure of pure n-hexane vapor was from 0 up to around 1 atm (0.1 MPa).

**Figure 1:**
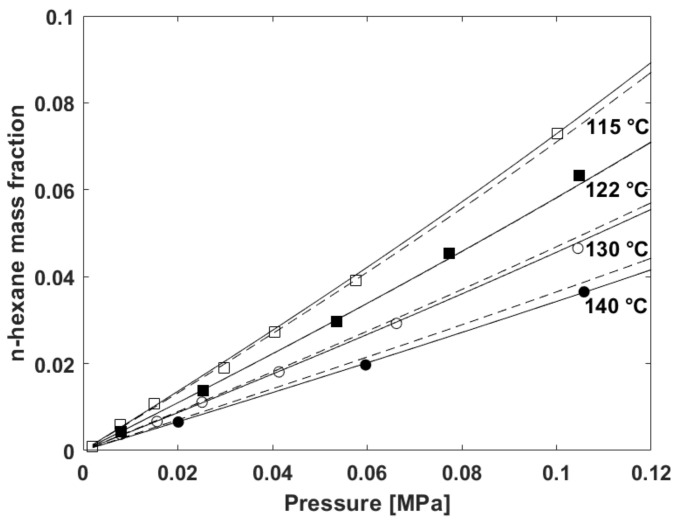
Experimental sorption isotherms of n-hexane vapor in V8880 at 115 °C (white squares), 122 °C (black squares), 130 °C (white circles), and 140 °C (black circles). Results of simultaneous best fitting of data by using Non Random Lattice Fluid (NRLF) model for mixtures with a temperature-independent binary interaction parameter are reported as dotted lines (best fitting value of *k*_12_ = −0.0754). Results of simultaneous best fitting of data by using NRLF model for mixtures using a *k*_12_ linearly dependent on temperature are reported as continuous lines (best fitting values of the two parameters are *k*_12,*a*_ = −0.1505 and *k*_12,*b*_ = 1.90 × 10^−4^ 1/K).

The results in terms of equilibrium sorption isotherms are reported in Figure 1. Data fitting by NRLF model for mixtures applied to the n-hexane/V8880 system is also reported and will be discussed in Section 5.2.

It is worth noting that three runs of “step-increase of pressure” experiments have been performed at each temperature. After a run made of several steps of increase of pressure of n-hexane vapor a total desorption on n-hexane was performed followed by another set of steps of increase of pressure. The average value of these measurements is reported at each pressure. As expected for rubbery polymer–penetrant mixtures, no hysteresis effect was noticed and all the data of the three runs performed at each temperature accommodate on a single isotherm.

Very few data are available in the literature to be compared with our results for sorption isotherms of n-hexane in polymer systems similar to the one under investigation. In particular, Francouer [38] reports results for n-hexane sorption in EPDM (i.e., terpolymers composed of ethylene, propylene, and various diene monomers). These data were collected in the 90–140 °C interval, although the exact temperatures at which data were collected are not specified for proprietary reasons, indicating simply T1 for highest temperature investigated and T5 for the lowest. These results compare well with those determined in the present investigation. In fact, the solubilities reported by Fracouer, expressed in terms of Henry’s constant, are around 0.008 phr/mbar (phr stands for grams of n-hexane per 100 g of polymer) in the lowest temperature range (presumably 90 to 115 °C) and about 0.0026 at 140 °C. In our case we have determined respectively, a value of 0.0081 phr/mbar at 115 °C and of 0.0029 phr/mbar at 140 °C. 

## 5. Discussion

### 5.1. Determination of NRLF Model Parameters for Pure Components

#### 5.1.1. Fitting of PVT Data of V8880 with NRLF EoS

As anticipated, on the basis of the information available on the molecular structure of the polymer, no specific self-interactions are expected to establish among groups on the polymer chains, so that a thermodynamic model has been used (NRLF) that does not contain any parameter related to specific interactions. The NRLF parameters determined by the best fitting procedure of the PVT behavior of pure polymer were only the three scaling parameters and the surface-to volume ratio, *s*. The fitting procedure performed by using the NRLF model has been applied exclusively to the data acquired above the melting temperature. The results of this procedure are reported in Figure S4 of the SI document and the best fitting values of model parameters for pure V8880 are reported in Table 1. The molecular weight of the polymer has been assumed to be 80,000 g/mol.

#### 5.1.2. Fitting of n-Hexane Vapor Pressure Data and Density Data at Vapor–Liquid Equilibrium

The values of the three scaling parameters of the NRLF model for the case of pure n-hexane were retrieved by a fitting procedure of data on vapor–liquid equilibrium at several temperatures. Furthermore, in this case, in view of the chemical structure of n-hexane, no specific interactions need to be accounted for. The surface to volume ratio, *s*, in this case was taken from the literature (see Table 1 for details). Results of the best fitting procedure using the NRLF model for pure compounds are reported in Figure S5 of the SI document and the estimated values of model parameters are reported in Table 1.

### 5.2. Fitting of Sorption Isotherms

Once the NRLF parameters of the two pure compounds have been retrieved, the NRLF model for mixtures has been implemented to interpret phase equilibrium between pure n-hexane vapor and binary n-hexane/V8880 mixtures. In view of the high molecular weight of the polymer molecules, the assumption that the polymer is not present in the external vapor phase has been made.

In view of the molecular structure of both polymer and n-hexane molecules, no self- or cross- hydrogen-bonding (or any other specific interaction) need to be accounted for and, as discussed, the NRLF model has been considered adequate to describe sorption isotherms.

To model sorption isotherms, besides the model parameters determined for pure V8880 and pure n-hexane, one additional parameter is still required, i.e., the polymer–penetrant mean field binary interaction parameter *k*_12_, which measures the departure of the mean field interaction energy of the binary mixture from the geometric mixing rule. This parameter is related to the couple of compounds involved in the binary mixture considered. In fact, based on the Lorentz–Berthelot combining rule for dispersive cross energy we have that [39]:

(21) $$\varepsilon_{ij}=(1-k_{ij})\sqrt{\varepsilon_{i}\varepsilon_{j}}$$

where $\varepsilon_{i}$ and $\varepsilon_{j}$ are the intersegmental interaction energies in the close-packed state for pure components ‘*i*’ and ‘*j*’, while $\varepsilon_{ij}$ is the intersegmental interaction energy (cross-interaction energy) in the close-packed state for between a segment of a molecule of component ‘*i*’ and a segment of a molecule of component ‘*j*’ in a mixture. The interaction parameter *k_ij_* is introduced to correct for the dispersion energies of unlike molecules. Other mixing rules have been proposed for asymmetric systems or in order to represent better the critical area. The value of the interaction parameter, *k_ij_*, is typically retrieved from fitting of experimental sorption isotherms, as we have done in the case at hand. However, some theoretical insight is useful to understand its physical origin. In fact, for relatively simple systems (e.g., mixtures of hydrocarbons or gases with hydrocarbons) the interaction parameter can be estimated from the following equation, derived from the Hudson–McCoubrey theory assuming the validity of Lennard-Jones potential [39]:

(22) $$k_{ij}=1-\left[2^{7}\left(\frac{{\left(I_{i}I_{j}\right)}^{1/2}}{\left(I_{i}+I_{j}\right)}\right)\left(\frac{\sigma_{i}^{3}\sigma_{j}^{3}}{{\left(\sigma_{i}+\sigma_{j}\right)}^{6}}\right)\right]$$

Here, *I_i_* and *I_j_* are, respectively, the ionization potentials of compound ‘*i*’ and compound ‘*j*’, expressed in eV. *σ_i_* and *σ_j_* are the diameters of segments of, respectively, molecules of type ‘*i*’ and of type ‘*j*’. The molecular-size diameters are expressed in Å. The Lennard-Jones value of the exponent in the attractive potential (*n* = 6) has been used in the previous equation. With some degree of approximation, Equation (22) can be restated as

(23) $$k_{ij}=1-\left[2\left(\frac{{\left(I_{i}I_{j}\right)}^{1/2}}{\left(I_{i}+I_{j}\right)}\right)\right]$$

Different expressions are obtained in the case of different interaction potentials. Based on these theoretical arguments, one would expect a limited temperature dependence of *k_ij_* and a stronger dependence on density (i.e., on mixture concentration). As reported in literature [40], it is important to assume the binary interaction parameter to be at least linearly dependent on temperature to adequately correlate liquid–liquid equilibria for polymers:

(24) $$k_{ij}\left(T\right)\;=k_{ij,a}+\;k_{ij,b}T$$

In the following we will interpret experimental sorption isotherms using two approaches: 

(i) using a temperature independent *k*_12_ (“athermal” assumption), whose value is obtained by a concurrent fitting of all the four experimental isotherms;

(ii) using of a *k*_12_ that is linearly dependent on temperature, whose value is again obtained by a concurrent fitting of all the four experimental isotherms.

- Use of a temperature independent binary interaction parameter

The value of the temperature independent binary interaction parameter has been retrieved by performing a one parameter concurrent best fitting with NRLF model of the four isothermal data sets for n-hexane sorption in V8880. Based on the fitting procedure, whose results have been already reported in Figure 1, we obtained the value *k*_12_ = −0.0754.

- Use of a temperature dependent binary interaction parameter

The four experimental isotherms were interpreted using the NRLF model with a *k*_12_ assumed to be linearly dependent on temperature:

(25) $$k_{12}\left(T\right)\;=k_{12,a}+\;k_{12,b}T$$

From concurrent fitting of the four sorption isotherms (see Figure 1) we have obtained the following values for the two fitting parameters:

*k*_12,*a*_ = −0.1505

*k*_12,*b*_ = 1.900 × 10^−4^ 1/K

It is noted from Figure 1 that the fitting quality is evidently improved at 130 and 140 °C as compared to the case of a temperature independent binary interaction parameter.

**Figure 2:**
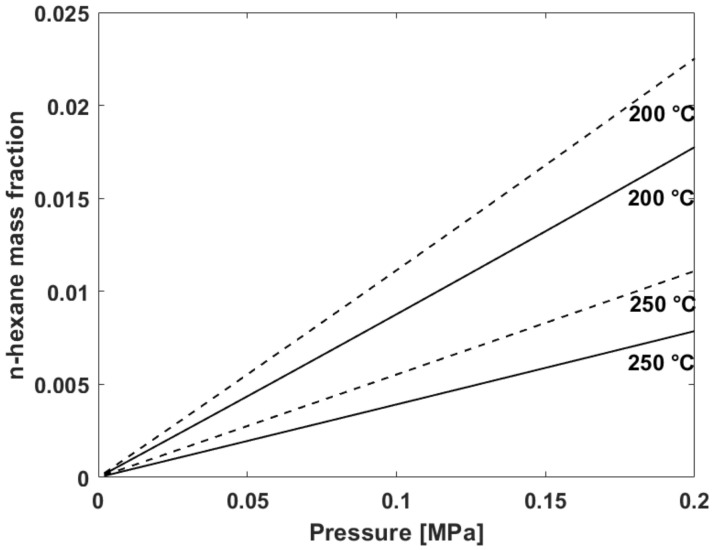
Comparison between the model prediction of n-hexane solubility isotherms in V8880 at T = 200 °C and T = 250 °C, carried out respectively assuming *k*_12_ independent on T (“athermal”, dotted line) and *k*_12_ linearly dependent on T (“linear”, solid line).

Once the values of the interaction parameters have been retrieved by fitting the experimental sorption isotherms at 115, 122, 130, and 140 °C, the NRLF model for mixtures, both in the case of temperature independent and of temperature dependent *k*_12_ values has been used to predict the sorption isotherms at 200 and 250 °C up to 0.2 MPa (2 atm), which are conditions of industrial interest, not accessible with the available experimental apparatus. In Figure 2, it is reported the comparison of the model predictions for n-hexane solubility isotherms at T = 200 °C and T = 250 °C, carried out respectively assuming *k*_12_ independent of T (i.e., athermal) and assuming *k*_12_ linearly dependent on T (i.e., “linear”). The predicted values of n-hexane mass fraction obtained using a linearly dependent binary interaction parameter are lower, at both temperatures, than those predicted by using a temperature independent value.

The physical motivation why we have used the NRLF approach is because compressible lattice fluid models are relatively simple theoretical frameworks well suited for the description of thermodynamics of mixture of solvents with rubbery/molten polymer [22] in the absence of specific interactions. The NRLF model is a reasonable compromise. In fact, it displays a somehow more complex structure as compared to the simpler SL theory [11,12,13], to account for non-randomness of contacts. However, differently from the SL model, it is thermodynamically consistent (see for a discussion on this point ref. [22]). However, the NRLF is not able to deal explicitly for the effect of a co-polymer structure. In fact, we have simplified the matter by treating the polymer as if it was a homo-polymer. As a consequence, the adopted model with the estimated parameters is only suited for the description of the specific system considered (V8880) and limited to its structural peculiarities.

Actually, some EoS based approaches have been proposed in the literature to tackle the challenging task of modelling the thermodynamics of mixtures of copolymers and solvents. A first attempt to extend EoS theories to the case of hetero-polymers, was performed by Panayiotou et. al., in the framework of a preliminary simplified formulation of a non-random compressible LF theory, [41]. This approach is suited for the case of block copolymers of two different unit types but is not adequate for different backbone structures as, for example, the case of alternate copolymers. Afterwards, Panayiotou et al. [42], developed a model for the case of random copolymers that was however limited to the prediction of the volumetric properties (EoS) of the pure copolymers.

More recently an alternative approach has been proposed by the authors of the perturbed-chain statistical associating fluid theory (PC-SAFT) [43] that have extended this model to the case of copolymers and their binary mixtures with low molecular weight penetrants [44]. In particular, their approach was applied to hetero-segmented molecules, displaying a well-defined alternating sequence of two repeating units or random sequences of blocks, interpreting sorption of n-pentane in an ethylene-propylene copolymer. 

Both the above mentioned LF and the PC-SAFT approaches need the introduction of ad hoc mixing rules for the evaluation of scaling parameters for the pure hetero-polymer based on the properties of the corresponding homo-polymers that take into account the composition in terms of types of unit segments and their statistical arrangement on the backbone. With the aim of describing the sorption thermodynamics of polymer–penetrant binary mixtures, it is then required the knowledge of three binary parameters, two polymer–penetrant and one polymer/polymer interaction parameters. Hence, a pre-requisite for using these approaches is a detailed knowledge of the chemical structure and of the type of arrangement, of the repeating units, which, as already mentioned, is not available for the industrial polymer of interest in the present investigation. Therefore, in the case at hand, the only feasible procedure consisted in the use of a reliable EoS model, treating the copolymer as an equivalent “fictive” homo-polymer.

### 5.3. Modelling n-Hexane Diffusivity

#### 5.3.1. Fitting Sorption Kinetics

**Figure 3:**
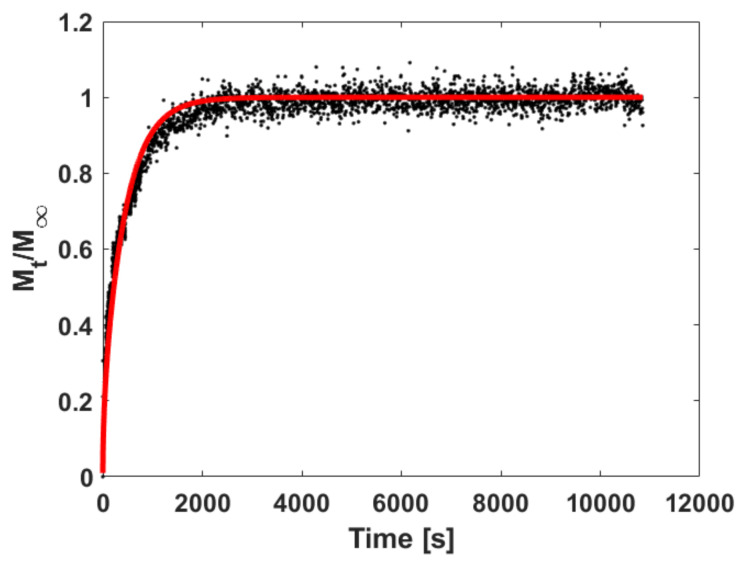
Fitting of sorption kinetics data of n-hexane for the pressure step from 405 to 576 mbar at 115 °C. Red continuous line is the result of data best fitting with Equation (19).

An example of the results of best fitting of the experimental sorption kinetics data with Equation (19) is reported in Figure 3 for the pressure step from 405 to 576 mbar at 115 °C. It is evident how the sample clearly attains a time-independent equilibrium value in the time frame of the experiment. The very good quality of fitting indicates that the assumption of Ideal Fickian behavior is well suited.

**Figure 4:**
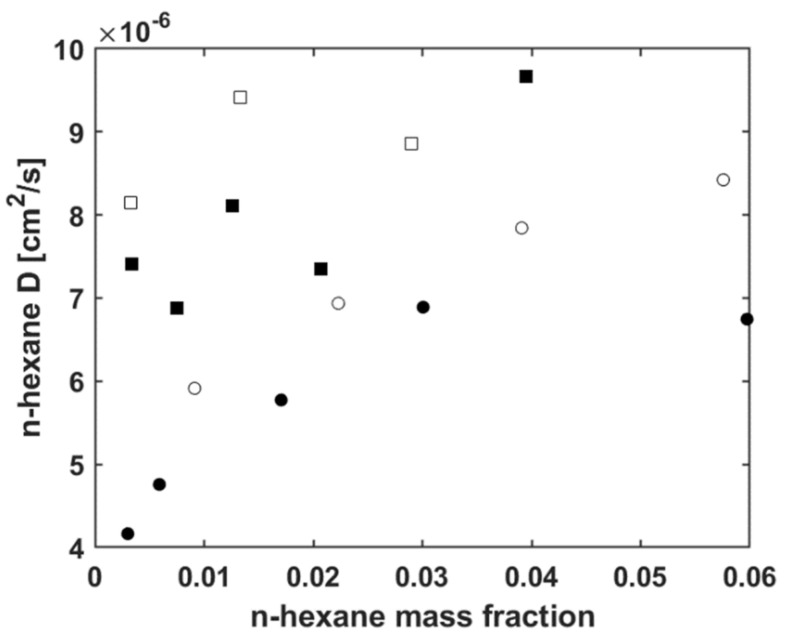
Values of n-hexane-V8880 mutual diffusivity as a function of n-hexane mass fraction in the polymer-penetrant mixture at the four investigated temperatures (115 °C (black circles), 122 °C (white circles), 130 °C (black squares), and 140 °C (white squares)).

The same fitting method has been applied for every pressure step at all the temperatures. In Figure 4 are reported the calculated values of n-hexane-V8880 Fickian mutual diffusivity (determined at the four investigated temperatures by the fitting procedure of sorption kinetics curves) as a function of the average mass fraction of n-hexane in each test. 

The values of mutual diffusivity evaluated at each incremental pressure step have been associated to the average value of n-hexane concentration within the sample during the sorption test, expressed as mass fraction in the polymer–penetrant mixture (calculated by making the arithmetic average of the initial and final values of n-hexane mass fraction at each pressure step). The values of *D* are affected by an error that is estimated to be ± 2 × 10^−7^ cm^2^/s.

In order to verify the reliability of the values determined for mutual diffusivity of n-hexane in V8880, we have compared these results with the very few data available in the literature for diffusion of n-hexane in similar polymer systems. 

In particular, data are available in the literature for n-hexane diffusion in EPDM [38]. These data were collected in the 90–140 °C interval, although the exact temperatures are not specified for proprietary reasons, indicating simply T1 for highest temperature investigated in that range and T5 for the lowest. The n-hexane mass fraction varied from 0 to 0.08. These results indicate that the diffusivity spans the range from 2 × 10^−6^ cm^2^/s (at the lowest temperature and concentration) to 6 × 10^−6^ cm^2^/s (at highest temperature and concentration). These values are in good agreement with our data. 

Further data are available in [45] for n-hexane diffusivity at 115 °C and at the zero concentration limit in ethylene–propylene elastomers. These authors determined a value of 2.241 × 10^−6^ cm^2^/s that compares well with the value of 3 × 10^−6^ cm^2^/s that one would estimate by extrapolating at zero concentration conditions our data collected at 115 °C.

#### 5.3.2. Fitting Sorption Kinetics

**Figure 5:**
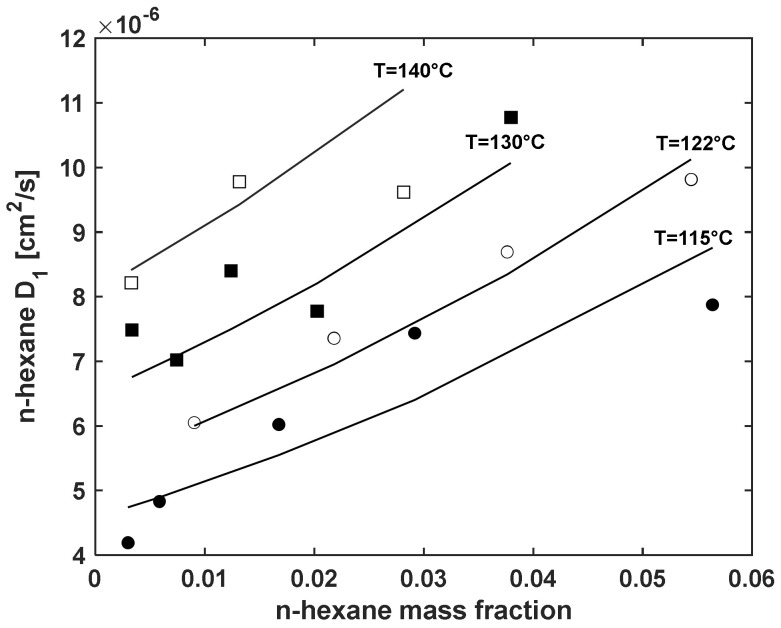
Values of *D*_1_ vs. mass fraction of n-hexane (linear plot) at the four investigated temperatures (115 °C (black circles), 122 °C (white circles), 130 °C (black squares), and 140 °C (white squares)). Continuous lines represent best fitting of data by means of Equation (26).

Using Equation (19), values of *D*_1_ can be readily obtained at the four investigated temperatures from the values of *D* and are reported in Figure 5. Based on the free volume theory of Duda and Vrentas [34,35], the following semi-empirical expression, for the penetrant intra-diffusion coefficient can be obtained:

(26) $$D_{1}=D_{00}\cdot \exp\left(\frac{E_{D}}{T}\right)\cdot \exp\left(E_{C}\omega_{1}\right)$$

Note that, in formulating Equation (26), explicit account of the effects of pressure has been disregarded due to the low values of pressures investigated. Here *D_00_*, *E_D_*, and *E_C_* represent the model parameters, which can be retrieved by a concurrent regression of the available values of *D*_1_ reported as a function of ω_1_, that represents the n-hexane mass fraction. Results of this best fitting procedure performed on the calculated values of *D*_1_ are reported in Figure 5. The optimized values of the parameters obtained are

*D_00_* = 5.57 × 10^−2^ (cm^2^/s)

*E_D_* = −3.653 × 10^3^ (K)

*E_C_* = 1.220

## 6. Conclusions

Mixture thermodynamics and mass transport properties of a POE/n-hexane system have been investigated experimentally and interpreted theoretically in view of their considerable interest to tailor conditions for processing operations involving this class of thermoplastic elastomers. 

The experimental results in terms of sorption equilibrium and diffusivities are valuable since very few experimental data are available in the literature. In particular, sorption isotherms of n-hexane in the industrial copolymer V8880 have been determined at several temperatures at sub-atmospheric pressures. Mutual diffusivities have also been determined at several temperatures and n-hexane concentrations and, from them, intra-diffusion coefficients of n-hexane in V8880 have been estimated. 

Modeling of sorption thermodynamics in co-polymers, like the one under investigation, is quite a challenging task and it is possible once the macromolecular structure is known. Since details on the structure of the investigated industrial copolymer were not available, the polymer has been treated as being a “fictive” homo-polymer approaching the modeling of sorption thermodynamics on the basis of a non-random lattice fluid theory, NRLF, developed for homo-polymers. This model provided a very satisfactory interpretation of the sorption isotherms producing an excellent concurrent fitting of the experimental results at several temperatures both with a temperature-dependent and a temperature-independent binary interaction parameter.

Dependences of mutual diffusivity of the POE/n-hexane mixture on temperature and concentration have been successfully interpreted using a semi-empirical model, based on free volume concepts, in terms of n-hexane intra-diffusion coefficient and of a thermodynamic contribution accounting for the composition dependence of n-hexane chemical potential.

The quantitative information provided by the thermodynamic and mass transport models developed here, once inserted in the equations that rule the evolution of the V8880/n-hexane system under processing, allow the adequate simulation and optimization of working conditions. 

## Figures and Tables

**Table 1:** NRLF parameters calculated by best fitting procedures.

Fluid	$\varepsilon_{h}^{*}$ (J × mol^−1^)	$\varepsilon_{s}^{*}$ (J × mol^−1^ × K^−1^)	$v_{sp,0}^{*}$ (cm^3^ × g^−1^)	*s*
n-hexane	3986.0	1.5009	1.2771	0.857 ^1^
V8880	4292.4	2.7679	1.1043	0.631

^1^ Taken from [27]. This “surface-to-volume” ratio, *s*, has been estimated in ref. [27] by using the widely adopted UNIFAC group contribution model [30].

## Data Availability

The data presented in this study and not reported in tables are available on request from the corresponding author.

## Supplementary Materials

The following are available online at https://www.mdpi.com/article/10.3390/polym13071157/s1, Table S1: Physical properties of VistamaxxTM 8880 [1], Figure S1: Schematic representation of GNOMIX high pressure dilatometer, Figure S2: Schematic representation of a McBain quartz spring sorption apparatus. The polymer sample hangs from the quartz spring located in the sorption chamber. (P: pressure transducer; GR: gas/vapor reservoir). Reprinted with permission from [3]. Copyright 2019 Elsevier B.V., Figure S3: Effective spring displacement during the experiment performed from 575.6 mbar to 1001.2 mbar at 115 °C. Equilibrium data are retrieved from the final constant displacement (rele-vant equilibrium data are highlighted by the green rectangle), Figure S4: Density vs. pressure isothermal data for V8880. Lines represent the results of simulta-neous best fitting of equilibrium dilatometric data using NRLF model. Temperature analyzed are, respectively, from top to bottom: 123.49 °C; 132.42 °C; 145.50 °C; 152.80 °C; 161.31 °C; 171.06 °C; 180.96 °C; 188.84 °C; 198.91 °C; 207.57 °C; 219.39 °C, Figure S5: n-hexane equilibrium data: (a) Vapor pressure data of n-hexane as a function of tem-perature; (b) temperature vs. density data for n-hexane at vapor–liquid equilibrium (black circles represent liquid density; white circles represent vapor density). Experimental points were retrieved from https://webbook.nist.gov/chemistry/ accessed on 26 February 2021. Results of simultaneous best fitting of data by using NRLF model are reported as continuous lines. References [17,22,36] are cited in the Supplementary Materials.
