# Supplementary material for: Modelling Sorption Thermodynamics and Mass Transport of n-Hexane in a Propylene-Ethylene Elastomer
Source: Polymers (Basel). 2021 Apr 4;13(7):1157. doi: 10.3390/polym13071157 (PMC8038456; doi:10.3390/polym13071157)
Supplement: Supplementary file 1 [file polymers-13-01157-s001.pdf]

polymers

Supplementary Materials

Modelling Sorption Thermodynamics and Mass Transport of n-Hexane in a Propylene-Ethylene Elastomer

Daniele Tammaro [1], Lorenzo Lombardi [1], Giuseppe Scherillo [1,2], Ernesto Di Maio [1,2], Navanshu Ahuja [3] and Giuseppe Mensitieri [1,2,*]

[1] Department of Chemical, Materials and Production Engineering, University of Naples Federico II, Piazzale Tecchio 80, 80125 Naples, Italy; daniele.tammaro@unina.it (D.T.); lorenzo.lombardi@unina.it (L.L.); gscheril@unina.it (G.S.); edimaio@unina.it (E.D.M.)
[2] UdR of Naples,, INSTM (National Interuniversity Consortium of Materials Science and Technology), Piazzale Tecchio 80, 80125 Naples, Italy
[3] Sulzer Chemtech Ltd., Else-Züblinstr. 11, 8404 Winterthur, Switzerland; navanshu.ahuja@sulzer.com
* Correspondence: mensitie@unina.it; Tel.: +39-081-768-2512

Citation: Tammaro, D.; Lombardi, L.; Scherillo, G.; Di Maio, E.; Ahuja, N.; Mensitieri, G. Modelling sorption thermodynamics and mass transport of n-hexane in a propylene-ethylene elastomer.. *Polymers* **2021**, *13*, 1157. https://doi.org/10.3390/polym13071157

Academic Editor: Francesco Paolo La Mantia

Received: 28 February 2021
Accepted: 29 March 2021
Published: 4 April 2021

Publisher's Note: MDPI stays neutral with regard to jurisdictional claims in published maps and institutional affiliations.

Copyright: © 2021 by the authors. Licensee MDPI, Basel, Switzerland. This article is an open access article distributed under the terms and conditions of the Creative Commons Attribution (CC BY) license (http://creativecommons.org/licenses/by/4.0/).

1. Materials and Techniques for Material Characterization and Testing

1.1. Materials

The investigated polymer is a commercial semicrystalline polyolefin elastomer (POE) made of isotactic propylene repeating units with random ethylene distribution, produced by ExxonMobil under the trade name Vistamaxx™ 8880. Its physical properties, available from the data sheet provided by the producer [1], are summarized in Table S1.

A reagent-grade n-hexane with a purity ≥ 99% was purchased from Sigma Aldrich, Milano, Italy.

Table S1. Physical properties of Vistamaxx™ 8880 [1].

Density (g/cm³)	Glass Transition Temperature (°C)	Melting Temperature (°C)
0.879	-22	97

1.2. Dilatometry

Specific volume of the V8880 elastomer, at equilibrium conditions, has been measured as a function of temperature and pressure in the following ranges: 25–200 °C, 10.0–200.0 MPa, to be used for the determination of NRLF model [2] parameters. The apparatus used to this purpose was a high-pressure dilatometer by GNOMIX (Gnomix Inc., Boulder, CO USA—see scheme of the apparatus reported in Figure S1). As received polymer granules, ca. 1 g, were included in a nickel cup and in the sample compartment of the cell to be subjected to isothermal tests with pressure step increase of 10.0 MPa and with 20 s waiting time before each measurement.

Since the GNOMIX equipment determines only volume changes, a reference absolute density measurement is required at known temperature and pressure conditions. To this aim, a Helium Pycnometer (AccuPyc II 1340, Micromeritics—Alfatest, Italy) was utilized to determine the density of the polymer at T = 25 °C and at P = 0.1 MPa.

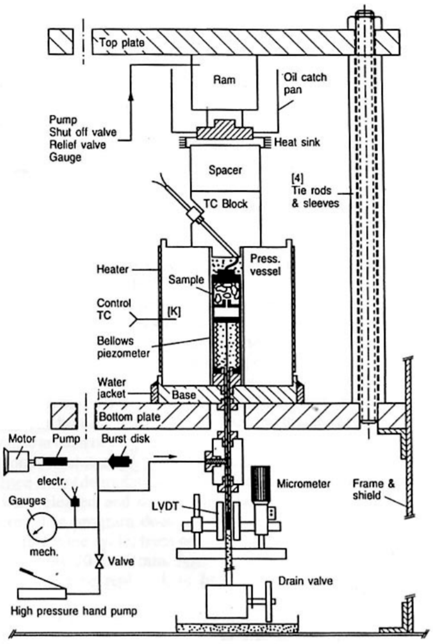

Figure S1. Schematic representation of GNOMIX high pressure dilatometer.

1.2. Gravimetric Sorption Tests

Vapor sorption experiments of n-hexane in V8880 were performed using a controlled atmosphere McBain micro-balance (see Figure S2), consisting in a quartz spring made of two counter-rotating elements (Ruska Instrument Co., Houston, TX, USA) located in a jacketed thermostated glass measuring cell. The weight increase of the polymer sample exposed to n-hexane vapor was determined from the measurement of the elongation of a quartz spring at which the sample was hanged. Sample was placed in a cylindrical pan that was suspended to the terminal hook of the spring. The thickness of the sample has been calculated from density data on the basis of the geometrical dimensions of the adopted cylindrical sample pan.

Service lines connect the thermostated measuring cell (accuracy of temperature control of the cell environment ± 0.05 °C) to a flask, to a pressure transducer (MKS Baratron 121A, with a full-scale of 1000 mbar, a sensitivity of 0.1 Torr, an accuracy of 0.1% of the reading) to the liquid pure n-hexane reservoir, to a turbomolecular vacuum pump and to the exhaust line. The spring was calibrated at three temperatures in the range 110–140 °C, to account also for the possible drift of the calibration constant with temperature. The adopted spring had a nominal elongation of 400 mm for 50 mg (with a sensitivity of $1.25 \cdot 10^{-4}$ mg/μm).

Spring elongation was continuously acquired using a travelling camera, with a microscope objective, that was fixed to a high accuracy motorized translation stage (V-817 by Physik Instrumente Karlsruhe, Germany) that was computer controlled, with a positioning accuracy of ± 2.5 μm. The measuring system, considering the characteristics of the adopted quartz spring and the accuracy of the translational stage, featured a minimum detectable weight change equal to $3.12 \cdot 10^{-4}$ mg. The video signal from the camera was continuously acquired and stored at prescribed time intervals in a computer. The whole measuring system (camera, moving stage) was controlled using a Labview® acquisition software by National Instruments, Austin, TX, USA.

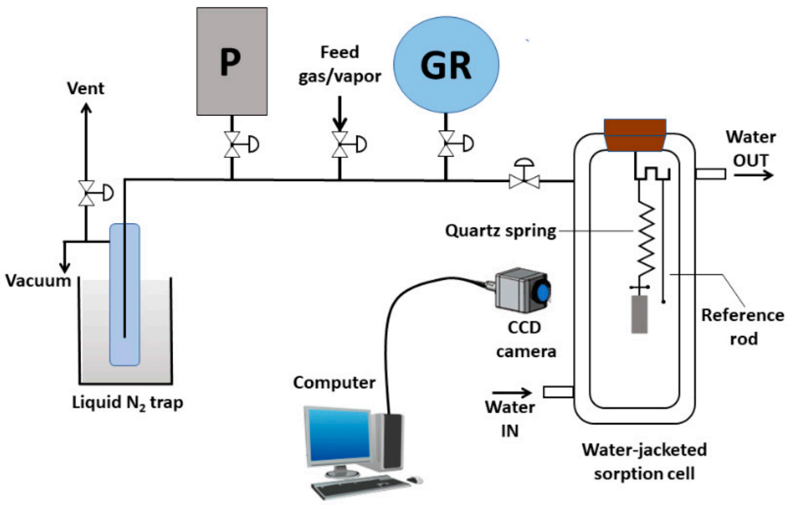

Figure S2. Schematic representation of a McBain quartz spring sorption apparatus. The polymer sample hangs from the quartz spring located in the sorption chamber. (P: pressure transducer; GR: gas/vapor reservoir). Reprinted with permission from [3]. Copyright 2019 Elsevier B.V.

Vapor sorption experiments were performed at 115, 122, 130, and 140 °C, at the pressure values of n-hexane vapor reported in Table S2. The determination of each sorption isotherm was repeated three times. As already mentioned, in this temperature range the polymer is above its melting temperature.

Table S2. Operating pressures (mbar) at the four different temperatures investigated.

T = 115 °C	T = 122 °C	T = 130 °C	T = 140 °C
20	80	80	201
79	254	156	597
151	536	251	1058
297	774	414	
405	1047	662	
576		1045	
1001			

As an example of a typical sorption run, it is reported in Figure S3 the sorption step performed from 576 mbar to 1001 mbar at 115 °C. The experiment start is at the instant of time at which n-hexane vapor enters the measuring cell. The displacement of the quartz spring end (where the sample is placed inside a quartz pan) is then followed as a function of time and recorded until the sorption equilibrium is attained. A time-independent equilibrium state is assumed to have been attained when the constant asymptotic value was maintained for a time that was at least twice the time needed to reach that value for the first time. In Figure S3 the entire experiment lasts about 5600 s, the time needed to reach the constant spring displacement being about 1800 s and the time waited at the plateau value about 3800 s.

From the spring displacement it was retrieved the increase of sample weight (corresponding to the weight of absorbed n-hexane) by using the spring weight/displacement calibration constant. The weight increase was then transformed in mass fraction of n-hexane absorbed in the polymer-n-hexane mixture (i.e. grams of n-hexane per gram of mixture). Buoyancy effects exerted by the n-hexane vapor have been taken into account by calculating the thrust of the vapor from the weight registered using the spring balance. The thrust has been estimated, for each sorption kinetics test, by extrapolating the weight

increase down to the starting time of each test. In addition, a remarkable, but physically sound, assumption that has been made is that no polymer is present in the vapor phase in view of the vanishingly small vapor pressure of the polymer.

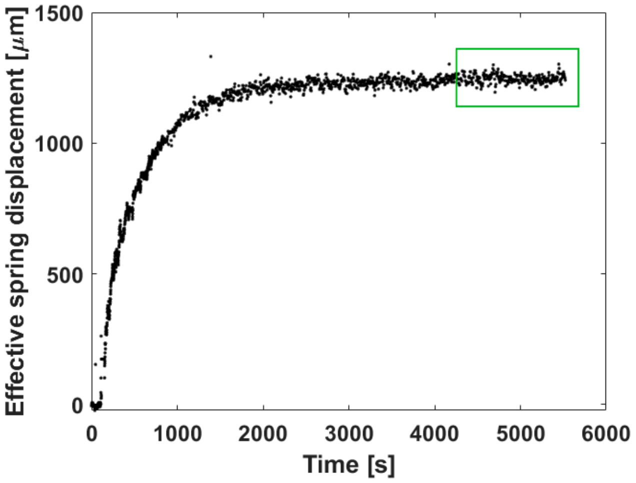

Figure S3. Effective spring displacement during the experiment performed from 575.6 mbar to 1001.2 mbar at 115 °C. Equilibrium data are retrieved from the final constant displacement (relevant equilibrium data are highlighted by the green rectangle).

2. Experimental Data Used for Determination of NRLF Parameters

2.1. Dilatometric (PVT) Behaviour of the V8880 Polymer

In Figure S4 are reported the outcomes of the experiments performed with the dilatometer in terms of density vs. pressure at different temperatures. The PVT data are reported in the pressure range 10–200 MPa and in the temperature range from 120 to 220 °C, since only data in this range have been used to retrieve the NRLF model parameters for pure V8880. Data fitting of PVT data was performed to determine the three scaling parameters of the model along with the molecular shape factor. Results of data fitting are also reported in Figure S4 and are discussed in Section 5.1 of the main manuscript.

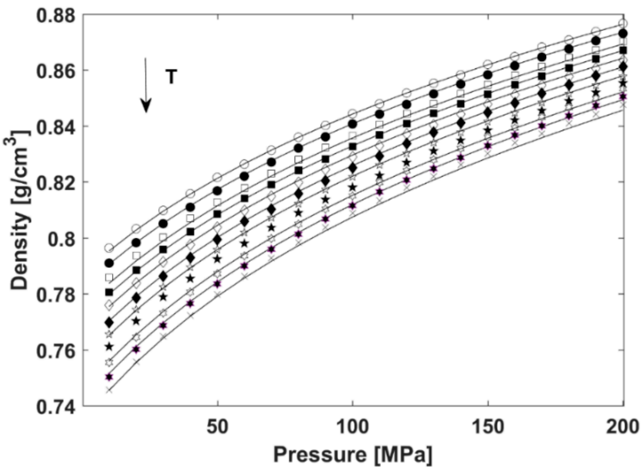

Figure S4. Density vs. pressure isothermal data for V8880. Lines represent the results of simultaneous best fitting of equilibrium dilatometric data using NRLF model. Temperature analyzed are, respectively, from top to bottom: 123.49 °C; 132.42 °C; 145.50 °C; 152.80 °C; 161.31 °C; 171.06 °C; 180.96 °C; 188.84 °C; 198.91 °C; 207.57 °C; 219.39 °C.

2.2. Equilibrium Data for Pure n-Hexane

Vapor pressure and equilibrium density data at liquid–vapor equilibrium for n-hexane were retrieved from thermodynamics databases (available online at the NIST website: https://webbook.nist.gov/chemistry/, accessed on 2 December 2020).

In Figure S5a are reported the vapor pressure vs temperature data at vapor-liquid equilibrium conditions. In figure S5b are reported temperature vs. equilibrium density data for the vapor phase (data on the left) and for the liquid phase (data on the right) at phase equilibrium. Data fitting of these equilibrium data was performed to determine the scaling parameters of NRLF model for pure n-hexane (see continuous lines in Figures S5a,b) as it will be discussed in Section 5.1 of the main manuscript.

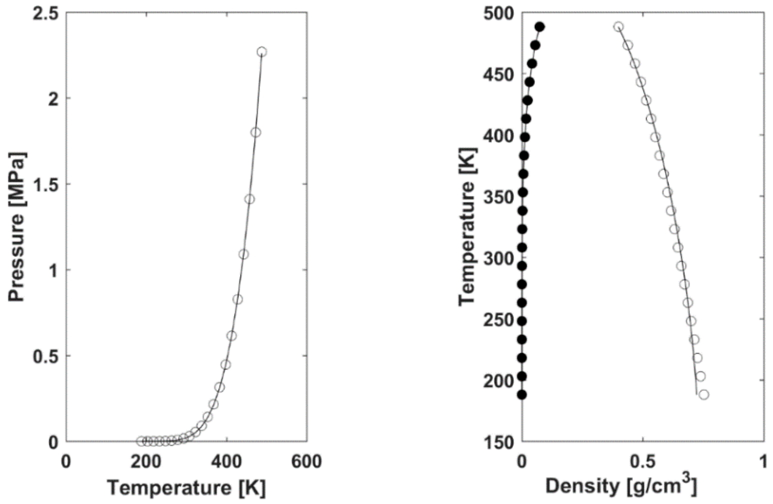

(**a**) (**b**)

Figure S5. n-hexane equilibrium data: (**a**) Vapor pressure data of n-hexane as a function of temperature; (**b**) temperature vs. density data for n-hexane at vapor–liquid equilibrium (black circles represent liquid density; white circles represent vapor density). Experimental points were retrieved from https://webbook.nist.gov/chemistry/. Results of simultaneous best fitting of data by using NRLF model are reported as continuous lines.

References

1. Product datasheet of Vistamaxx™ Performance polymer 8880 (propylene elastomer). Available online: http://exxonmobilchemical.com/ (accessed on 14 july 2020).
2. Panayiotou, C.; Pantoula, M.; Stefanis, E.; Tsivintzelis, I.; Economou, I.G. Nonrandom Hydrogen-Bonding Model of Fluids and Their Mixtures. 1. Pure Fluids. *Ind. Eng. Chem. Res.* **2004**, *43*, 6592-6606.
3. Mensitieri, G.; Scherillo, G.; Panayiotou, C.; Musto, P. Towards a predictive thermodynamic description of sorption processes in polymers: the synergy between theoretical EoS models and vibrational spectroscopy. *Mat. Sci. Eng. R* **2020**, *140C*, 100525.
